# Comprehensive Evaluation of the Efficiency of Yeast Cell Wall Extract to Adsorb Ochratoxin A and Mitigate Accumulation of the Toxin in Broiler Chickens

**DOI:** 10.3390/toxins12010037

**Published:** 2020-01-07

**Authors:** Suvi Vartiainen, Alexandros Yiannikouris, Juha Apajalahti, Colm A. Moran

**Affiliations:** 1 Alimetrics Research Ltd., 02920 Espoo, Finland; j.apajalahti@alimetrics.com; 2 Alltech Inc., 3031 Catnip Hill Road, Nicholasville, KY 40356, USA; ayiannikouris@Alltech.com; 3 Alltech SARL, Rue Charles Amand, 14500 Vire, France; cmoran@alltech.com

**Keywords:** ochratoxin A, mitigation, mycotoxin binding, yeast cell wall extracts

## Abstract

Ochratoxin A (OTA) is a common mycotoxin contaminant in animal feed. When absorbed from the gastrointestinal tract, OTA has a propensity for pathological effects on animal health and deposition in animal tissues. In this study, the potential of yeast cell wall extracts (YCWE) to adsorb OTA was evaluated using an in vitro method in which consecutive animal digestion events were simulated. Low pH markedly increased OTA binding to YCWE, which was reversed with a pH increased to 6.5. Overall, in vitro analysis revealed that 30% of OTA was adsorbed to YCWE. Additional computational molecular modelling revealed that change in pH alters the OTA charge and modulates the interaction with the YCWE β-d-glucans. The effectiveness of YCWE was tested in a 14-day broiler chicken trial. Birds were subjected to five dietary treatments; with and without OTA, and OTA combined with YCWE at three dosages. At the end of the trial, liver OTA deposition was evaluated. Data showed a decrease of up to 30% in OTA deposits in the liver of broilers fed both OTA and YCWE. In the case of OTA, a tight correlation between the mitigation efficacy of YCWE between in vitro and in vivo model could be observed.

## 1. Introduction

Ochratoxin A (OTA), a mycotoxin produced by toxigenic species of *Aspergillus* and *Penicillium* fungi, is a common contaminant in cereals, fruits, and nuts intended for human and animal consumption. For both these fungal species, moisture and temperature are key factors for growth and toxin production. Thus, OTA can be produced during plant growth, harvest, and storage [1,2]. The European Commission provides maximum guidance levels for OTA in animal feeds. For broiler chickens, the OTA concentration in complete feed and in individual cereal ingredients used for feed formulation should not exceed 0.1 and 0.25 mg/kg, respectively [3]. Nevertheless, the presence of OTA at a chronic concentration, whilst not necessarily posing a health issue, could be disadvantageous to bird performance and as such contribute to a negative economic output in a broiler operation. In this context, the use of appropriate mitigation programs is often recommended. Toxin adsorbents are frequently supplemented to animal diets to prevent the deleterious effect of mycotoxins, among them specific yeast cell wall extracts have shown promising results [4,5,6]. 

Several experiments were carried out to study the effects of OTA on production parameters in animal husbandry. Studies have shown that administration of diets contaminated with OTA reduces feed intake and body weight gain in broiler chickens in a dose-dependent manner and negatively affects weight gain in swine [7]. OTA has been shown to exert toxicity by inhibition of mitochondrial function, increased oxidative stress, and inhibition of protein synthesis [8]. At a molecular level, OTA pathology includes damaged membrane lipids, DNA mutations, and nitrosylated proteins [8]. In both mammalian and avian animals, sub-acute exposure to OTA has nephrotoxic, immunosuppressive, and teratogenic effects [9]. Carcinogenic effects of OTA have been demonstrated in mice and rats; while in humans, renal and testicular cancer have been associated with OTA exposure [9]. 

To date, absorption of OTA has been studied mainly in mice and rats. The results for rats indicate that the major sites of OTA absorption are the duodenum and proximal jejunum, but the toxin is also absorbed from the stomach [10]. Studies of OTA absorption show that the toxin can be quickly detected in peripheral blood, with the maximum concentration in blood following oral administration of OTA being reached after 20 min in chickens, 1 h in rabbits, and 10 h in pigs [11]. Absorbed OTA binds to blood proteins, such as albumin, and distributes throughout the animal. The half-life of OTA in blood after oral administration depends greatly on animal species, e.g., it is 6.7 h in quail, 39 h in mice, 120 h in rats, and 510 h in Rhesus monkeys (*Macaca mulatta*) [12]. OTA is mainly detoxified into ochratoxin α (OTα) by animal tissues and by intestinal anaerobic bacteria. Other metabolites of OTA are formed in the liver by cytochrome P450 and these metabolites differ from one animal species to another [13]. OTA and OTα are excreted in faeces, whereas other OTA metabolites, together with OTA and OTα, are found in urine [14].

Mycotoxin adsorbents or sequestrants are used in animal husbandry due to the common occurrence of mycotoxins in feedstuffs. The purpose of the mycotoxin adsorbents is to bind to the toxin in the digestive tract of the animal, after which the adsorbent-toxin complex is transported intact through the gastro-intestinal tract of the animal and excreted. In poultry, adsorbents such as yeast cell walls, esterified glucomannan, and aluminosilicate have been tested [15]. For the toxin adsorbent to be functional, it is essential that the digestive processes of the animal do not alter its properties. In order to analyse the effects of digestion, toxin, and adsorbent have been subjected to digestion treatments—such as low pH—in in vitro conditions, and the results compared with a control without digestion treatment [16,17].

Yeast cell wall extract (YCWE) has been studied with emphasis on its toxin adsorbent properties [18]. The YCWE has been shown efficacious in sequestering mycotoxins in in vitro and decreasing the adverse effects of mycotoxins in vivo conditions [15,16]. In the current study, the adherence of YCWE to OTA were examined both in vitro and in silico. The aim was to obtain accurate information on the interaction between OTA and YCWE during digestion treatments and on the effect of low pH on OTA-YCWE complex formation. Unlike previous studies, the effectiveness of YCWE in decreasing the OTA bioavailability was subsequently assessed in vivo in broiler chickens, at a dose that did not exceed the recommendation for commercial broiler feeds presented in the European Union guidance [3]. In accordance with the EFSA dossier preparation instructions for Substances for the Reduction of Mycotoxin Contamination (SMRC) [19], the efficacy of YCWE in reducing bioavailability of OTA was determined with analysis of OTA deposited in broiler liver tissue. 

## 2. Results

### 2.1. In Vitro Evaluation of the Influence of Simulated Digestive Conditions on OTA Sequestration

**Figure 1:**
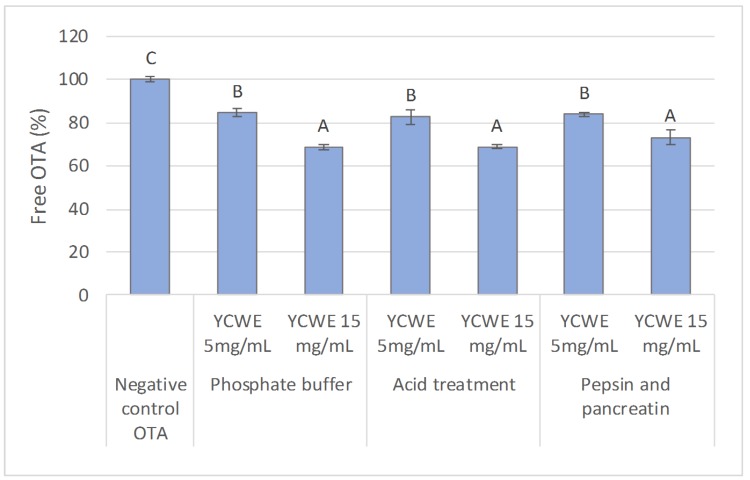
Percentage of free ochratoxin A (OTA) measured in the supernatant after pH adjustment to 6.5 following reaction in a two-step digestive simulation (acidic and two-step pepsin and pancreatin enzymatic treatment) with yeast cell wall extract (YCWE) used at 5.0 and 15.0 mg/mL inclusion level. Error bars indicate standard error. Different letters above bars indicate significant differences between treatments (*p* < 0.05).

When radiolabelled OTA was incubated with YCWE at pH 6.5, there was a significant decrease in the level of free OTA compared to the control treatment without YCWE. The level of free OTA decreased by 17.1 ± 0.5% (SE) with a YCWE dose of 5 mg/mL and by 28.4 ± 0.6% with a YCWE dose of 15 mg/mL. Subjecting the YCWE solution to further digestion treatments—i.e., low pH—and a 2-step pepsin and pancreatin enzymatic treatment, did not alter the binding properties of YCWE to OTA measured at pH 6.5, as shown in Figure 1. This suggests that the YCWE product tested was not susceptible to the digestive treatments.

### 2.2. In Vitro OTA Sequestration by YCWE

**Figure 2:**
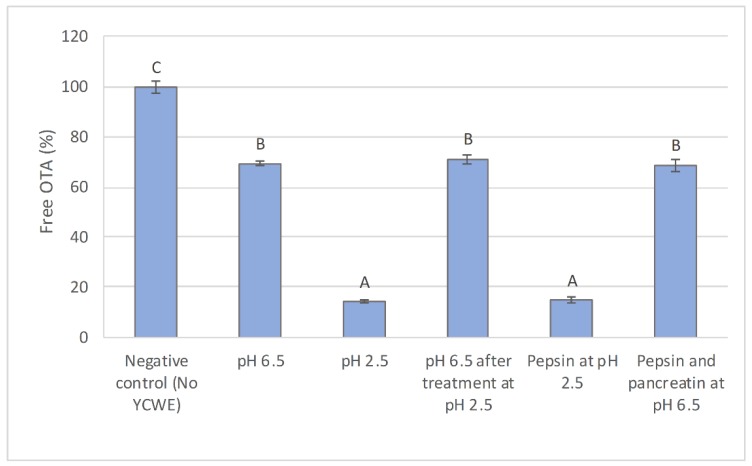
Percentage of free ochratoxin A (OTA) measured after the interaction with yeast cell wall extract (YCWE), at each individual digestion phases and evaluation of the influence of pH on sequestration. The YCWE inclusion level was 15 mg/mL. Error bars indicate standard error. Different letters above bars indicate significant differences between treatments (*p* < 0.05).

Evaluation of the effect of pH in each digestion phase on YCWE sequestering activity toward OTA revealed a major decrease in the level of free [^3^H]-OTA activity at pH 2.5, implying that the binding efficacy was increased in an acidic gastric environment (Figure 2). Further analysis revealed that the decrease in free [^3^H]-OTA level depended only on the pH lowering step and was reversible, as the level of free toxin increased following pH neutralization to 6.5. None of the treatments with digestive enzymes had any additional effect on the detected [^3^H]-OTA activity.

### 2.3. In Silico Assessment of the Sequestration Properties Investigated by Molecular Mechanics

**Figure 3:**
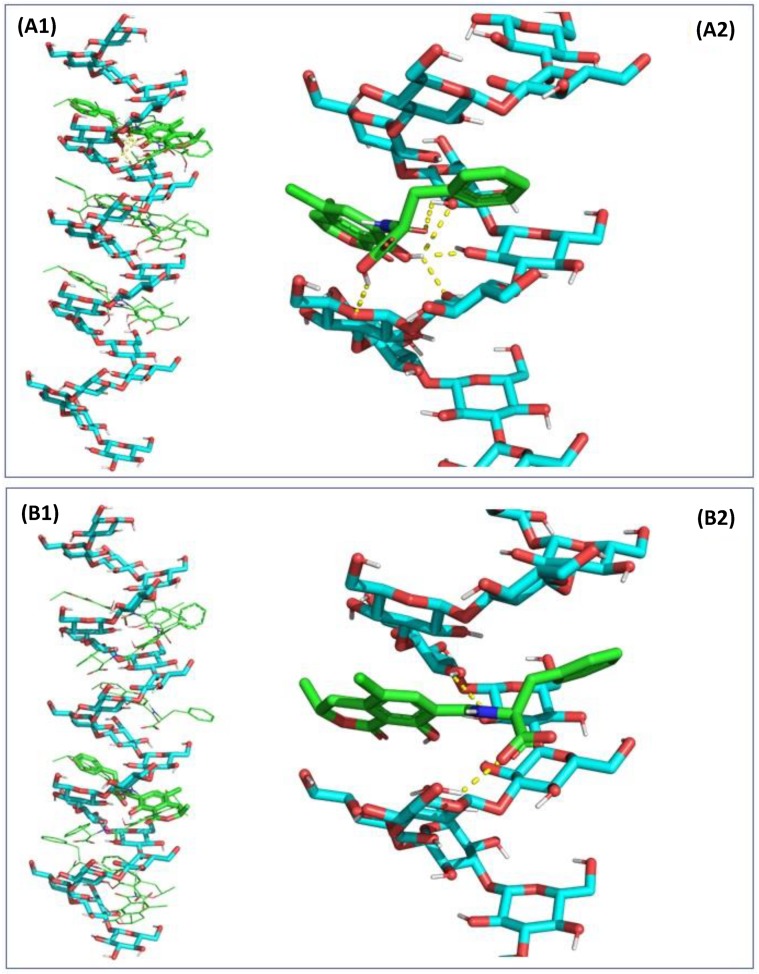
Computer generated views of the energy-minimized 9 states (**A1**,**B1**) of ochratoxin A (OTA) docking into β-(1,3)-d-glucans chain alone with (**A2**) corresponding to the most energy favorable OTA docking with no charge (corresponding to charge state at pH 3.0) and (**B2**) corresponding to the most energy favorable OTA docking with a partial charge equal to −1 (corresponding to charge state at pH 6.5).

**Figure 4:**
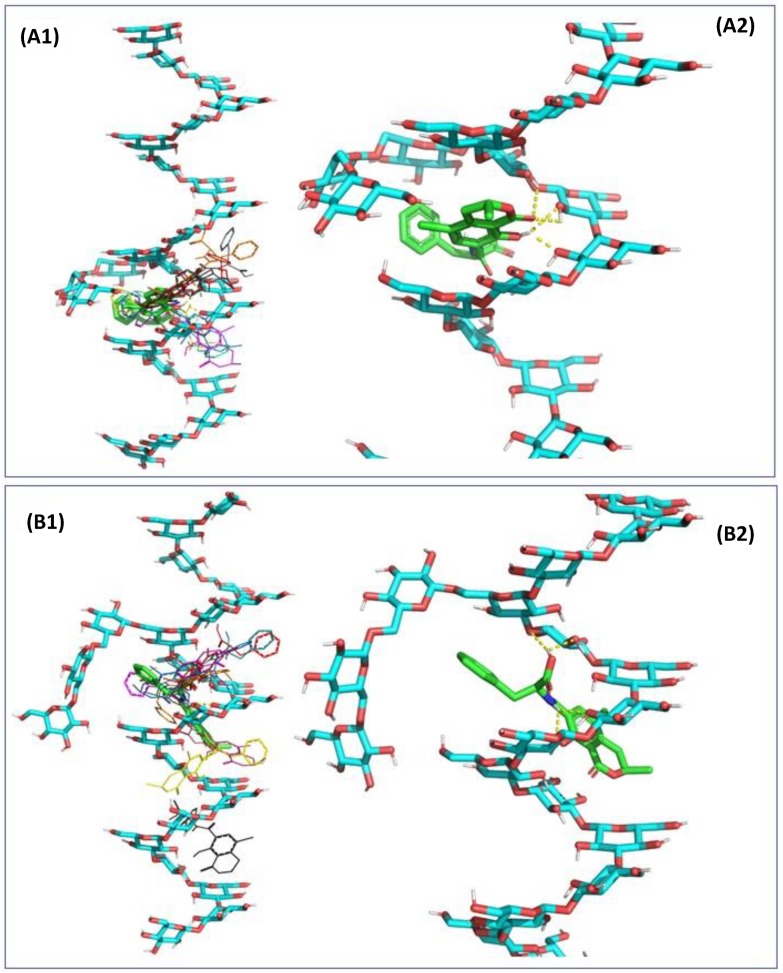
Computer generated views of the energy-minimized 9 states (**A1**,**B1**) of ochratoxin A (OTA) docking into β-(1,3)-d-glucans either branched with the first conformation of β-(1,6)-d-glucans (**A2**, most energy favorable OTA docking position) and the second conformation of β-(1,6)-d-glucans (**B2**, most energy favorable OTA docking).

Molecular mechanics modelling and docking were performed under vacuum and demonstrated that, because OTA possesses five rotatable bonds mainly located around the amide bond connecting the dihydroisocoumarin moiety to l-phenylalanine, it could adapt to a larger set of docking sites with little energetic penalties of 3 kcal/mol of amplitude. In the docking experiment performed, parallel positioning of these two moieties (Figure S1A1,A2) gave the lowest binding energy and thus accounting for the highest affinity of interaction (Table S1), whereas an orthogonal positioning of the two moieties was less favourable. Nevertheless, the extension of the interaction to the entire chain of β-d-glucans demonstrated that OTA can be positioned in several adjacent binding sites along the β-(1,3)-carbohydrate chain (Figure 3) forming up to three hydrogen bonds. The presence of side chains of β-(1,6)-d-glucans provided increased stability of interaction, with the majority of the most favourable poses centered around the β-(1,3)/β-(1,6)-d-glucans pocket (Figure 4), especially in the case of a compact side chain conformation (Figure 4B1,B2). The OTA molecule has a five hydrogen bond acceptor count and three donor count, and the strongest acidic pKa was of 3.17. Under the conditions investigated in vitro and in vivo, the uncharged form of OTA represented 82.50% of the OTA population at pH 2.5 whereas at pH 6.5, 96.45% of the OTA population consists of a single charged deprotonated molecule on the carboxylic moiety, with a charge of −1.03. An increase of pH to 8.5 would produce a second charge on the hydroxyl group of the coumarin moiety. The β-D glucan carbohydrate has its strongest acidic pKa for a value of 11.22, making this a fully uncharged molecule for values of pH under pH 9.0. The changes in protonation of the OTA molecule were evaluated in terms of docking interaction, but both states exhibited somewhat comparable binding affinities (Table S1). The orientation of the l-phenylalanine moiety was further influenced by the presence of side chains of β-(1,6)-d-glucans which could involve further π-stacking interactions than protonation changes. 

### 2.4. In Silico Assessment of the Sequestration Properties Investigated by Molecular Dynamics

**Figure 5:**
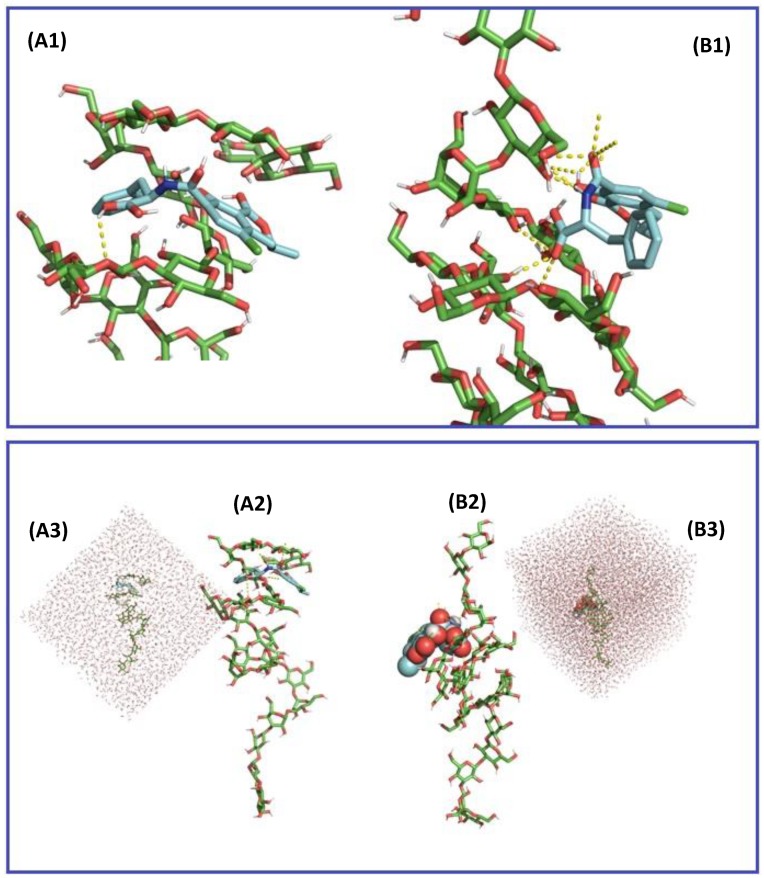
Computer generated views of the molecular interaction following a molecular dynamic simulation in a solvent box containing 8172 molecules of water, with a singly deprotonated molecule of ochratoxin A accounting for its state at a pH of 2.5 and when docked according to its highest affinity pose into an unconstrained (**A1**, interaction site detail, **A2**, full molecules displayed, **A3**, full molecule within solvent box displayed) and constrained (**B1**, interaction site detail, **B2**, full molecules displayed, **B3**, full molecule within solvent box displayed) conformation of β-(1,3)-d-glucans chain. The generated views corresponded to a 10-ns molecular dynamic simulation equilibrated at 310 K.

**Figure 6:**
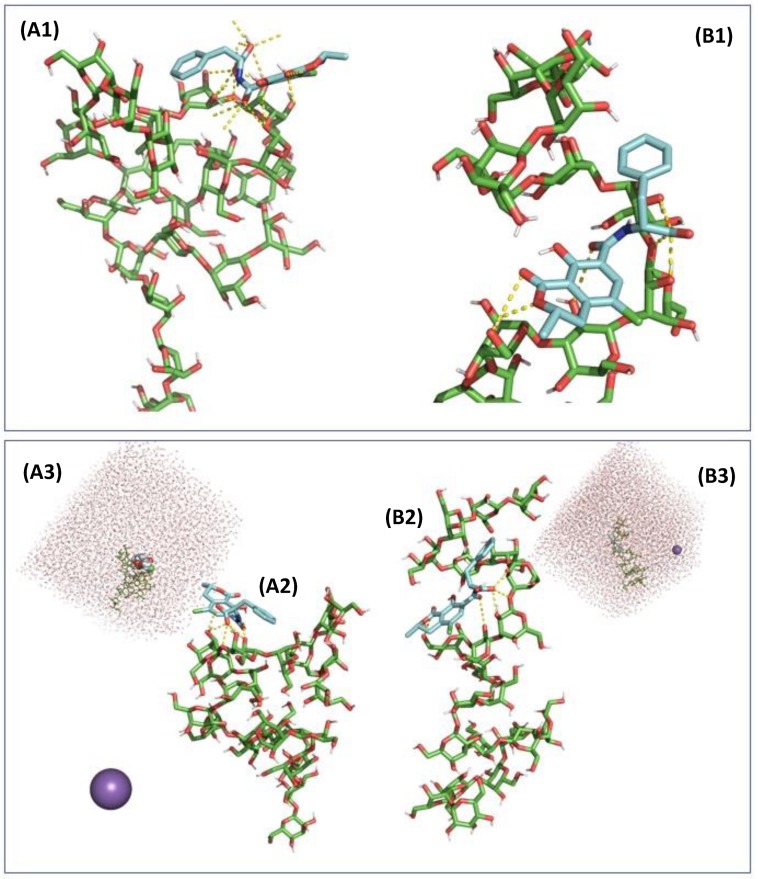
Computer generated views of the molecular interaction following a molecular dynamic simulation in a solvent box containing 8171 molecules of water and 1 Na^+^ ion, with a singly deprotonated molecule of ochratoxin A accounting for its state at a pH of 6.5 and when docked according to its highest affinity pose into an unconstrained (**A1**, interaction site detail, **A2**, full molecules displayed, **A3**, full molecule within solvent box displayed) and constrained (**B1**, interaction site detail, **B2**, full molecules displayed, **B3**, full molecule within solvent box displayed) conformation of β-(1,3)-d-glucans chain. The generated views corresponded to a 10-ns molecular dynamic simulation equilibrated at 310 K.

To understand the chemical interaction of OTA and glucans further, we carried out molecular dynamics simulation to understand the stability of the interaction in a solvated system using the best pose of OTA in the β-(1,3)-d-glucan chain as determined with the molecular mechanics investigations. To study the impact of pH, if it is possible to introduce ions in the solvent box environment it is only useful for the extreme ends of the pH scale due to the limited size of the solvent box and ion to water molecule ratio. A better approach was to evaluate the strength of interaction by calculating all relevant pKa values for the molecules of interest and assign their dominant protonation state at the two pH studied herein. In this context, if β-(1,3)-d-glucans have a very high pKa and do not exhibit any charges under the conditions tested, OTA was studied either fully protonated or singly deprotonated corresponding to the protonation stages at pH 2.5 and 6.5, respectively. In this context, after neutralization and a short minimization step, a molecular dynamics simulation was carried out. The results showed that under simulation conditions, the β-(1,3)-d-glucans structure was affected, and the positioning of the OTA could then move outside the β-(1,3)-d-glucans binding pocket, which was particularly the case at pH 6.5. Two models were tested with and without constraints on the glucan chain to work with two degrees of conformational changes of the carbohydrate. In a non-constrained receptor setting, the interaction between the β-(1,3)-d-glucans and the fully protonated OTA exhibited a higher incidence of neutral interaction (Lennard-Jones potential energy) than a singly unprotonated OTA, with energy values of −114.7 and −47.4 kJ/mol respectively, whereas coulombic potential energy accounting for ionic interactions was, as expected, more important for the singly deprotonated OTA state, −47.5 and −104.4 kJ/mol (Table 1). In this context, the total potential energy of interactions favoured the protonated OTA stage and the observed increased stability at pH 2.5 compared to pH 6.5. In a restraint β-(1,3)-d-glucan conformation, if Coulombic energy was of the same order of magnitude for the two stages of protonation of OTA, neutral stability interactions were increased for the singly deprotonated OTA, increasing the overall total stability of the complex at pH 6.5. For both β-(1,3)-d-glucan conformations, average hydrogen bonds for unprotonated OTA was around 3, whereas it varied between 1 and 2 for the protonated OTA. Interaction with water was more pronounced with the deprotonated OTA, inducing a higher degree of competition between the glucan receptor and the solvent environment. The computer generated views at pH 2.5 (Figure 5) and at pH 6.5 (Figure 6) showed that in acidic condition, both unconstrainted and constrained β-(1,3)-d-glucan conformations tended to have their helical shape preserved and could maintain the protonated OTA in the docking site. Under neutral pH 6.5 conditions, the β-(1,3)-d-glucan structure was heavily affected and the position of OTA tended to move outside of the binding pocket site, the binding of the deprotonated OTA molecule becoming more of a surface interaction, prone to higher interaction with water molecule of the solvent box.

### 2.5. In Vivo Broiler Chicken Feeding Trial

Feeding broilers with OTA at the maximum concentration recommended under European Commission guidance did not affect any of the broiler performance parameters measured (Table 2), e.g., no differences were observed in body weight, feed consumption (not shown), or mortality. Moreover, FCR was unaffected by OTA and YCWE addition (Table 2). Thus, administration of OTA and YCWE to broilers for 14 days was well tolerated by the birds.

### 2.6. Analysis of OTA Deposits in Broiler Livers

**Figure 7:**
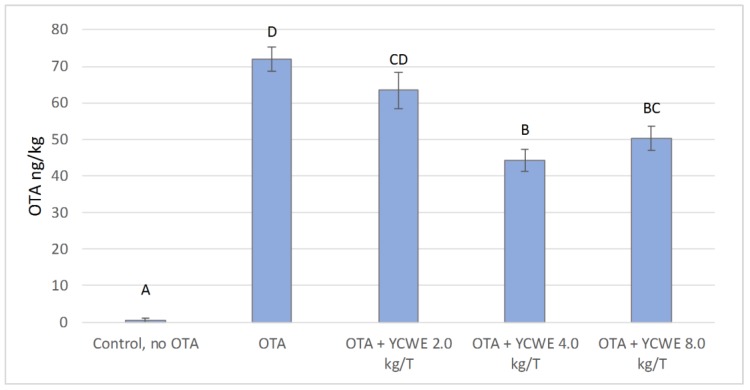
Deposition of ochratoxin A (OTA) in the liver of broiler chickens expressed in ng/kg following the dietary administration of yeast cell wall extract (YCWE) at varying inclusion rates. Error bars indicate standard error of the mean. Different letters above bars indicate significant differences between treatments (*p* < 0.05).

Feeding broiler chickens the maximum recommended concentration of OTA led to toxin deposition in the liver (Figure 7). The toxin deposition rate was highest in birds fed OTA alone, while adding YCWE to the diet lowered the level of OTA in the liver. In broilers fed the control diet without OTA, the level of OTA was negligible. Adding YCWE to the diet at doses of 4.0 and 8.0 kg/T significantly reduced deposition of OTA in the liver compared with birds on diets without YCWE addition. When the datasets from the in vitro trial at pH 6.5 and the feeding trial were compared, it was observed that in both experiments YCWE addition decreased OTA by a similar fraction from the maximum detected level (Figure 2 and Figure 7).

## 3. Discussion

Functional feed additives, such as toxin adsorbents, must remain active throughout the digestive tract of the animal and as such evaluating their integrity and susceptibility to the digestive environmental condition is necessary. Therefore, the YCWE product tested in this study was subjected to chemical, enzymatic, and physical digestion treatments simulating in vitro broiler chicken digestion. Tests of the toxin sequestration properties of YCWE after each digestion step revealed no significant changes in the level of radioactive OTA bound to YCWE, indicating that YCWE functionality and activity are preserved in the gastro-intestinal tract of broilers. Measuring changes in radioactivity is a highly sensitive method for assessing alterations in the concentration of radiolabelled compound (tritium-labelled OTA was selected as a signal molecule when assaying in vitro product efficacy). The method allowed analysis of the experimental samples directly after one centrifugation step, thus avoiding more laborious extraction procedures, isolation, and detection methods.

Analysis of the adsorption of OTA to YCWE during simulated consecutive digestion steps revealed a substantial increase in adherence at low pH. At pH 2.5, more than 80% of OTA was found to have bound to YCWE, whereas the enzymatic digestion treatments tested (pepsin, pancreatin) had no effect on binding performance. However, when the pH was re-adjusted to 6.5, OTA release from YCWE was observed. In broiler chickens, it has been shown that through reverse peristalsis digesta can also occasionally move from distal to proximal digestive tract. Thus, it is possible that digesta moves back from neutral duodenum to acidic gizzard [20]. Although our model did not specifically mimic such reverse peristalsis, we showed that when changing pH from neutral to acidic, and again to neutral, OTA binding was reversible, which is to say it becomes bound and is then released again. Thus, our model enables also predicting such a situation. This observation shows similarities with results obtained by Oh et al. [17], where low pH in combination with YCWE was found to decrease the toxicity of OTA in cultured macrophages. Oh et al. [17] suggest that the mechanism of decreased toxicity involved conformational changes in OTA at pH below 5.0, which in turn affected hydrogen bonding and van der Waals forces between OTA and YCWE. The increased adherence of OTA to YCWE at pH 2.5 and the detachment of OTA at pH 6.5 observed in the present study may support the mechanism suggested by Oh et al. [17]. However, it appears from our in silico studies that the changes in OTA conformation tended to happen independently of pH, due to several degrees of liberty from the presence of seven rotatable bonds around the amide bond. The observed changes in affinity could then be attributed rather to protonation level of the molecule than OTA conformational changes. The OTA molecule has a carboxylic acid group with pKa1 at 4.4 [21] and, according to our result, has a strong acidic pKa of 3.17. This functional group increasingly occurs in protonated form at pH lower than the pKa1 and protonation affects the molecule, making it electrochemically uncharged and decreasing its polarity. When pH is increased up to pH 6.5, OTA has a theoretical charge of −1.03 involving a deprotonation of the carboxyl group. If changes in the molecular structure due to deprotonation are minimal compared to protonated form (Figure S1), the glucan conformational structure can be dramatically affected, as demonstrated by the molecular dynamics simulation using two protonation states of OTA (Figure 5 and Figure 6, Table 1), as well as the stability of interaction that involves in its majority, the carboxylic group of OTA in the formation of hydrogen bonds. All these factors may have an effect on OTA adherence to YCWE and demonstrated that the changes in β-(1,3)-d-glucan organization could transform the docking occurring at pH 2.5 into a surface interaction at pH 6.5. Moreover, the deprotonated form of OTA was more prone at interacting with the solvent environment, with an increased amount of H-bond formed, that directly competed with the capacity of the molecule at binding to the YCWE. 

In the broiler trial, no acute OTA toxicity occurred in the birds, since all measured growth parameters, including mortality, were similar between the treatment groups. The dose of OTA fed to the broiler chickens was 0.1 mg/kg of feed, which is the maximum recommended level in commercial broiler feed in the European Union. In earlier studies, decreased body weight gain has been reported for broilers fed OTA at a dose of 4 mg/kg [22] or 0.5–0.25 mg/kg [23,24]. However, literature data on body weight changes are not conclusive, as no change in body weight gain was observed in a study by Politis et al. [25] in which 0.5 mg/kg of OTA was fed to broilers for 42 days. Therefore, the finding that the low dose of OTA applied in the present study did not change broiler performance parameters was expected and is in line with the previous research. However, these findings apply to a single mycotoxin and it is hypothesised that when several toxins are present at the same time, even if their levels are within the statutory or guidance limits, there may be synergistic forms of toxicity, as demonstrated by the impact of OTA in conjunction with the presence of penicillic acid (*Penicillium* spp. mycotoxin) in a macrophage culture assay [26] and negative effects on animal performance. In such cases, toxin binders may significantly improve the performance of production animals. 

Analysis of the liver of broilers fed an OTA-containing diet for 14 days showed clear accumulation of OTA in liver tissues. It is thus evident that OTA is deposited in broiler liver tissues even when the dose of toxin is present in feed below the guidance level of the European Commission. However, the concentration of OTA in liver samples (0.072 ± 0.003 µg/kg) was low relative to the maximum recommended level of 2–10 µg/kg in products intended for human consumption in the European Union and Canada [27]. Several-fold higher OTA concentrations than were found in the present study have been detected in broiler products and chicken eggs in Pakistan, where 41% of broiler products have been found to be contaminated with OTA and the OTA concentration in broiler liver is as high as 3.56 µg/kg [28]. As the concentration of OTA in broiler chicken tissues is a result of toxin concentration in feed [29,30] it is clear that OTA concentrations exceeding 0.1 mg/kg are present in broiler feeds in some geographical regions. 

When the broiler chickens in the present study were fed both YCWE and OTA, the OTA deposits in the liver decreased significantly. This decrease in OTA deposition was dependent on the dose of YCWE. The optimal YCWE dose tested was 4.0 kg/T, because the concentration of OTA detected in broiler liver was found the lowest with this dose. Lowered OTA deposition in the liver has also been reported in previous studies in which broiler chickens were fed OTA-contaminated feed together with toxin-deactivating products such as *Trichosporon mycotoxinivorans* [29,30] plant extracts [31], and yeast products [32]. However, in all those studies the concentration of OTA in broiler feed was at least five-fold higher than in the present study. Thus, a novel finding in the present study is that OTA deposition can be reduced using YCWE at chronic level of exposure of OTA. A low level can still be potentially damaging, e.g., in a study by Pozzo et al. [33], an OTA level of 0.1 mg/kg feed did not induce clinical signs but was shown to induce mild histological pathology in immune organs.

In the present study, a clear correlation was observed between results from in vitro and in vivo studies. The relative decrease in free OTA in the adsorption models used herein was comparable to the reduction in OTA concentration found in broiler livers. Dose dependence was observed both in vitro and in vivo, as an increase in the YCWE dosage decreased the measured OTA concentration. Previous studies have revealed a specific dose dependency in the fraction of OTA deposited in the liver, e.g., in a study by Hanif et al. [29] using mycotoxin deactivators, the effect of the deactivators was greater when the OTA dosage in broiler feed was increased. In the present study, there was an indication of a similar effect with the increase in YCWE inclusion from 2.0 to 4.0 kg/T but no difference was observed with a further increase of the level inclusion up to 8.0 kg/T. At 4.0 kg/T, the highest measured OTA concentration in the feed, OTA deposition in the liver was the lowest observed in the study, apart from the control. Thus, addition of YCWE to animal feed has the potential to decrease OTA deposition in the liver.

## 4. Conclusions

Overall, this work provides a methodological and practical insight to the demonstration of the binding properties of YCWE, showing an ability to chemically interact with OTA, dependent on the digestive physiological conditions and pH. This was demonstrated in vitro and by both biochemical and computational mechanistic/dynamic experiments. As reported here, changes in pH can induce conformational changes not only of the OTA molecule but also of the β-d-glucans, the bioactive component of YCWE, affecting the geometry of the binding site and consequently the affinity of interaction. A complementary in vivo study demonstrated that YCWE, when fed to broiler chickens at different inclusion levels, could tightly correlate these in vitro/in silico observations and mitigate the impact of OTA by significantly reducing accumulation in liver tissue. Collectively, these results showed that YCWE could sequester OTA and prevent its deposition in liver, which in turn can protect the animal from a mycotoxin challenge when present at low and chronic levels in the diet. 

Mycotoxins, including OTA, are highly prevalent in animal feed. A three-year survey by Rodrigues et al. [34] found that up to 93% of feed samples were contaminated with OTA, while other mycotoxins, such as aflatoxin and fumonisin, were similarly common. As a consequence, correct harvest and storage methods are essential in controlling mycotoxin contamination of feedstuffs [35]. However, as this study indicates, including mycotoxin adsorbents or deactivators provides further assurance of good animal health and diminished toxin presence in human foodstuffs.

## 5. Materials and Methods 

### 5.1. In Vitro Assessment of OTA Sequestration by YCWE

Yeast cell wall extract (YCWE) (Mycosorb A+^®^, Alltech Inc., Nicholasville, KY, USA) used in the study was added to 50 mM Na-phosphate buffer (pH 6.5) in silyated test vials (Supelco, Sigma-Aldrich, Saint Louis, MO, USA) in a final reaction volume of 10 mL. Buffer alone was used as a control. A mixture of tritium ([^3^H])-labelled OTA (Moravek Biochemicals, Inc., Brea, CA, USA) and non-radiolabeled OTA (AppliChem GmbH, Darmstad, Germany) was prepared in Na-phosphate buffer to give a final OTA concentration of 20 ng/mL and [^3^H] activity of 0.08 µCi/mL. The OTA solution was added to test treatment vials (see treatment condition below) for a final YCWE concentration of 5 and 15 mg/mL. Reaction vials were mixed and incubated at +37 °C for 2 h on a rotary shaker (GFL, Burgwedel, Germany). After incubation, the vials were centrifuged for 10 min at 3000× *g*. The [^3^H] activity of unbound [^3^H]-OTA was analysed by taking a 50 µL sample of the supernatant, adding it to 3.5 mL of scintillation cocktail (OptiPhase SuperMix, Perkin Elmer, Waltham, MA, USA) and measuring the [^3^H] activity using a scintillation counter (Microbeta 1450, Perkin Elmer, Waltham, MA, USA).

### 5.2. In Vitro Digestive Simulation Conditions and OTA Sequestration Activity of YCWE

In the first in vitro model experiment, YCWE at two final concentrations (5 and 15 mg/mL) was subjected to three successive environmental conditions before the resultant adsorption efficacy was calculated based on the measure of the amount of free OTA remaining in the supernatant: (1) a 50 mM Na-phosphate buffer environment maintained at pH 6.5, considered as the control treatment; (2) a simulated gastric environment initiated by adjusting the pH to 2.5 with 1 M HCl and reacting with YCWE for 3 h at +37 °C, which represented the acid alone gastric digestion treatment; (3) a simulated enzymatic digestion treatment in an initial conditioning step through the addition of pepsin (Sigma-Aldrich, Saint Louis, MO, USA) at 7.5 mg/mL under the same environmental conditions as with (2), followed by a second step of pH adjustment to 6.5 with 1 M NaOH and addition of 2.5 mg/mL of pancreatin (Sigma-Aldrich, Saint Louis, MO, USA), representing the concomitant digestive enzymatic treatment. YCWE was incubated during the two successive enzymatic digestion steps at +37 °C for 3 h on a rotary shaker. After incubation in the pH was adjusted to 6.5 and the free OTA was measured.

In the second in vitro model experiment, the adsorption efficacy of YCWE (15 mg/mL) was evaluated based on the amount of free OTA remaining in the supernatant after each stage of digestive simulation: (1) starting stage, Na phosphate buffer maintained, pH 6.5; (2) first intermediate stage, after the acid gastric digestion treatment at pH 2.5; (3) second intermediate stage, after acid gastric digestion and neutralisation at pH 6.5; (4) third intermediate stage, after the one-step enzymatic (pepsin) treatment at pH 2.5; (5) final stage, after the two-step enzymatic (pepsin followed by pancreatin) treatment at pH 6.5. 

In both experiments, a mixture of OTA (final concentration 20 ng/mL, [^3^H] activity 0.08 µCi/mL) was added to the digested YCWE and non-digested control. The activity of unbound [^3^H] OTA was measured in five replicates following the above described assessment of OTA sequestration by YCWE. The pH adjustments between digestive phases were monitored, and the indicated values represent the actual pH measurements. 

### 5.3. In Silico Assessment of the Sequestration Properties Investigated by Means of Molecular Mechanics and Dynamics

Molecular mechanics investigations were carried out using several open-source programs: (1) Constructs of the β-(1,3)-d-glucan chain branched with a side chain of β-(1,6)-d-glucan previously generated on a Silicon Graphics computer with the Accelrys package (formerly Accelrys, now Biova Dassault Systemes, San Diego, CA, USA) in CFF91 force-field with steepest descent minimization [36]. This structural assembly was considered in the present study as the receptor, with three distinct conformations being investigated, a β-(1,3)-d-glucan chain alone, and two different β-(1,6)-d-glucan branched β-(1,3)-d-glucan chain conformations, as modelled in previous work; (2) OTA three-dimensional structure was downloaded from Chemspider compound repository under the permalink record 390954 (Royal Society of Chemistry, Burlington, VT, USA, http://www.chemspider.com/Chemical-Structure.390954.html) with OTA considered as the ligand in our study; (3) Molecular docking experiments were run on AutoDock Vina (The Scripps Research Institute, La Jolla, CA, USA, [37]). Before starting the docking process, all molecules were added with their polar hydrogens and were evaluated for their rotatable bonds. Seven degrees of liberty were found on the particular OTA molecule. The receptor conformation was constrained to the previously minimized conformation [38]. A grid box with a spacing of 60.0 Å in every x, y, z direction was parameterized and centered on a site of interaction at the proximity of the β-(1,3)- and β-(1,6)-d-glucans branching but also including in the grid box dimension adjacent binding sites over the entire single helical chain length consisting of 36 glucopyrannose +/− 3 side chain residues. PDB extension files were converted into pdbqt files before performing docking using Autodock Vina (v1.5.6, The Scripps Research Institute, La Jolla, CA, USA), an empirical scoring function that calculates affinity. The docking experiment was performed under C++ generic programming in a Microsoft Windows^®^ (10 Pro, 1809, Microsoft Corporation, Redmond, WA, USA) environment and produced a maximum number of nine binding modes with a maximum energy range of 3 kcal/mol, and for which an affinity (kcal/mol) and distances from the best modes were measured. 

Molecular dynamics were investigated using several open-source programs: (1) doGlycans (v1.0, Open Source program under GNU General Public License, v3.0, Free Software Foundation Inc, Boston, MA, USA), a tool to prepare carbohydrate structures under an Optimized Potentials for Liquid Simulations for All Atoms (OPLS-AA) modified force-field and related topology files for simulation [39] was used under Python 3 script and the Conda management system (Anaconda Inc., Austin, TX, USA) applied to the β-d-glucans receptor; (2) TopolGen (v1.1, Open Source Program under GNU General Public License, v3.0, Free Software Foundation Inc., Boston, MA, USA), a Perl script, and LigParGen (Open Source Program,http://zarbi.chem.yale.edu/ligpargen/) were used to produced GROMACS-formatted topology from the above generated PDB files docking experiment, compatible with an OPLS-AA force-field for OTA [40,41,42]; (3) GROMACS 2019.1 (GROMACS, Open Source Program under GNU General Public License, v3.0, Free Software Foundation Inc., Boston, MA, USA, www.gromacs.org, [43]) molecular dynamics simulation package was used to simulate receptor-ligand complex over 10 ns in a neutralized solvated system (water) at 37 °C and to study the initiation of the interaction energy at different charge states corresponding to a pH simulation performed at pH 3.0 and 6.5 to match our in vitro evaluations [44,45]. OTA was restrained using a force constant of 1000 kJ mol^−1^ nm^−2^ while the constrained and unconstrained structure of β-d-glucans were evaluated. Interaction energy was evaluated according to ionic coulomb energy potential and Lennard-Jones neutral molecule interaction potential energy expressed in kJ/mol.

PyMOL (The PyMOL Molecular Graphics System, v2.2.3 Schrödinger, LLC., New York, NY, USA) was used for visualization of the 3D molecular structures for all stages of molecular mechanics and molecular dynamics simulations. The chemical properties of OTA were calculated using the Chemicalize Chemoinformatic platform (ChemAxon, Cambridge, MA, USA, http://www.chemaxon.com) for the calculation of pKa of the molecules and chemical properties at different pH values.

### 5.4. In Vivo Dietary Treatments

The trial was conducted with a basal diet (Table 3) that was used as a negative control and also to generate the mycotoxin challenge dietary treatments. For these latter treatments, pure OTA (Sigma-Aldrich, Saint Louis, MO, USA) was dissolved in ethanol and mixed into a small amount of broiler feed. This mixture was step-wise mixed into larger batch of feed and eventually to final OTA challenge diet. The different doses of YCWE were added to small batches of broiler feed which were then mixed into final dietary treatments.

### 5.5. Animal Trial

The feeding trial was conducted in the research facility of Alimetrics Ltd. in Southern Finland, in accordance with EU Directive 2010/63/EU. Following the standard operating procedures of Alimetrics Ltd., ethical approval or animal trial permit was not required as the substance under investigation is an approved feed ingredient in the EU and the level of Ochratoxin A included in the diets were below EU guidance levels (European Commission, 2006, [3]). 240 newly hatched male Ross 508 broiler chicks were randomly allocated to five feeding treatments divided between 40 pens (8 pens per treatment, 6 birds per pen). Temperature and lighting programs followed the Aviagen recommendations for Ross broilers (Aviagen Group, Huntsville, AL, USA). For the first 7 days, all birds were fed a basal diet (Table 4). From day 8 onwards, the birds on the control diet continued a basal diet while the other treatment groups received a targeted 0.090 mg/kg OTA-contaminated diet added to three varying amounts of YCWE (2.0, 4.0, 8.0 kg/T). The birds were individually weighed on days 1 and 21. Feed conversion ratio (FCR) was corrected for mortality by including the weight of deceased birds in the calculation. FCR was calculated over the period 1–21 days.

### 5.6. Mycotoxin Analysis of Dietary Treatments

Confirmation of the levels of OTA in final feed via analysis (Table 4). Basal diets were also analysed for their content in aflatoxin B1 and B2, deoxynivalenol, nivalenol, zearalenone, fumonisins B1 and B2, T-2 toxin, and HT-2 toxin by means of LC-MSMS (Nutricontrol, Veghel, The Netherlands). Considering the measurement uncertainty announced by Nutricontrol, we can conclude that the content of OTA in the diets did not significantly differ from each other.

### 5.7. OTA Analysis of Liver Tissues

Livers were collected from four broilers per pen. The livers were combined in pairs, resulting in two analytical samples per pen. The tissues were ground and stored at −20 °C until analysis. OTA analysis was performed by extracting 5.0 g of ground liver with 25 mL of 1% NaHCO_3_: methanol (30:70, v/v). After centrifugation, 10.0 mL of the supernatant was mixed with an equal volume of PBS and applied onto an OchraTest WB immunoaffinity column (Vicam, Milford, MA, USA). The effluent was filtered through a 0.45 µm syringe filter and analysed by high pressure liquid chromatography equipped with a fluorescent detector (HPLC Prominence, Shimadzu, Kyoto, Japan). The HPLC separation was performed by means of a Phenomenex Luna C18(2) 3 µm, 150 × 4.60 mm column equipped with a Gemini C18, 4 × 3 mm SecurityGuard pre-column (Phenomenex Inc., Torrance, CA, USA) at a flow rate of 0.8 mL/min. Injection volume was set to 40 µL and column oven temperature 30 °C. Fluorescence detection was carried out at an excitation wavelength of 333 nm and emission wavelength of 460 nm. The limit of detection measured from standard deviation of background signal was 0.4 ng/kg and the limit of quantification was 2 ng/kg.

### 5.8. Data Analysis

Statistically significant differences between mean values of the parameters tested were analyzed with ANOVA using Tukey’s honestly significant difference (HSD) post-hoc test in the SPSS statistical software package (IBM, version 22, Armonk, NY, USA).

## Figures and Tables

**Table 1:** Interaction energy measured as short-range Coulombic potential energy (ionic interaction), short-range Lennard-Jones potential energy (neutral molecules interaction) and total energy (kJ/mol) after molecular dynamic simulation, in a two conformation of β-(1,3)-d-glucans unconstrained or constrained using a force constant of 1000 kJ/mol/nm^2^, in a solvent box (water) when interacting with two protonated stages of ochratoxin A (OTA).

pH	OTA State	Energy	(kJ/mol)	Average H-Bond G3-OTA	Average H-Bond OTA-Water
**Constrained Receptor**					
pH 2.5	Protonated OTA and glucan chain Energy	Coulombic Energy	−47.5541 ± 3.0	2.218	3.861
		Lennard-Jones energy	−114.6890 ± 3.9		
		Total	−162.2431		
pH 6.5	Singly deprotonated OTA and glucan chain energy	Coulombic Energy	−104.4470 ± 4.6	3.010	5.624
		Lennard-Jones energy	−47.3817 ± 4.4		
		Total	−151.8287		
**Unconstrainted Receptor**					
pH 2.5	Protonated OTA and glucan chain Energy	Coulombic Energy	−58.1255 ± 2.4	0.911	4.316
		Lennard-Jones energy	−87.1747 ± 2.9		
		Total	−145.3002		
pH 6.5	Singly deprotonated OTA and glucan chain energy	Coulombic Energy	−101.9410 ± 9.2	2.891	6.604
		Lennard-Jones energy	−80.0512 ± 6.7		
		Total	−181.9922		

**Table 2:** Broiler chicken performance in the 21-day feeding trial with ochratoxin A (OTA) and yeast cell wall (YCWE) added to the diet (*p* < 0.05), FCR = feed conversion ratio.

Diet	Body Weight (g)		Mortality (%)	FCR Day 1–21
	Day 1	Day 21		
Control	45.9 ± 0.7	856 ± 33	2.1 ± 2.1	1.62 ± 0.12
+ OTA	46.5 ± 0.6	841 ± 37	4.2 ± 4.2	1.48 ± 0.03
+ OTA + YCWE 2.0 kg/T	45.0 ± 0.6	782 ± 22	6.3 ± 3.0	1.73 ± 0.15
+ OTA + YCWE 4.0 kg/T	45.6 ± 0.5	832 ± 22	2.1 ± 2.1	1.49 ± 0.05
+ OTA + YCWE 8.0 kg/T	46.0 ± 0.9	832 ± 45	2.1 ± 2.1	1.50 ± 0.04

**Table 3:** Composition of the basal diet fed to broiler chickens for 21 days.

Ingredient	%
Wheat	60.31
Soybean meal	31.60
Rapeseed oil	4.0
Monocalcium phosphate	1.70
Limestone	1.30
NaCl	0.40
Mineral premix ^1^	0.20
Vitamin premix ^2^	0.20
Methionine	0.10
Lysine	0.09
Threonine	0.10
Total	100.00

^1^ Containing: calcium 296.8 g/kg; iron 12.5 g/kg; copper 4 g/kg; manganese 25 g/kg; zinc 32.5 g/kg; iodine 0.225 g/kg; selenium 0.1 g/kg. ^2^ Containing: calcium 331.3 g/kg, vitamin A 6,000,000 IU, vitamin D3 2,250,000 IU; vitamin E 30,000; tocopherol 27,270 mg/kg; vitamin K3 1505 mg/kg; vitamin B1 1257.3 mg/kg; vitamin B2 3000 mg/kg; vitamin B6 2009.7 mg/kg; vitamin B12 12.5 mg/kg; biotin 75 mg/kg; folic acid 504 mg/kg; niacin 20,072 mg/kg; and pantothenic acid 7506.8 mg/kg.

**Table 4:** Amounts of ochratoxin A (OTA) and yeast cell wall (YCWE) added to the experimental diets.

Diet	Target Dose of OTA (mg/kg)	Analysed Dose of OTA (mg/kg) ^1^
Control	0.0	<0.001
+ OTA	0.090	0.110
+ OTA + YCWE 2.0 kg/T	0.090	0.099
+ OTA + YCWE 4.0 kg/T	0.090	0.140
+ OTA + YCWE 8.0 kg/T	0.090	0.110

^1^ OTA analysis performed at NutriControl (Veghel, The Netherlands). The reported repeatability and reproducibility of the analysis was 20% and 25%, respectively.

## Supplementary Materials

The following are available online at https://www.mdpi.com/2072-6651/12/1/37/s1, Figure S1: Computer generated views of the aligned energy-minimized nine states of ochratoxin A (OTA) docking into β-(1,3)-d-glucans chain alone (A) with A1 corresponding to OTA with no charge (corresponding to charge state at pH 3.0) and A2 with a partial charge equal to −1 (corresponding to charge state at pH 6.5); into β-(1,3)-d-glucans branched with β-(1,6)-d-glucans conformation 1 (B); and conformation 2 (C). In these views, only the OTA is represented to account for conformational changes of the ligand molecule; Table S1: Energy values describing the affinity of interaction between ochratoxin A (OTA) and three different β-d-glucans conformations found in yeast cell wall extract.
