# Supplementary material for: Comprehensive Evaluation of the Efficiency of Yeast Cell Wall Extract to Adsorb Ochratoxin A and Mitigate Accumulation of the Toxin in Broiler Chickens
Source: Toxins (Basel). 2020 Jan 7;12(1):37. doi: 10.3390/toxins12010037 (PMC7020489; doi:10.3390/toxins12010037)
Supplement: Supplementary file 1 [file toxins-12-00037-s001.pdf]

Supplementary Materials: Comprehensive Evaluation of the Efficiency of Yeast Cell Wall Extract to Adsorb Ochratoxin A and Mitigate Accumulation of the Toxin in Broiler Chickens

Suvi Vartiainen *, Alex Yiannikouris, Juha Apajalahti and Colm A. Moran

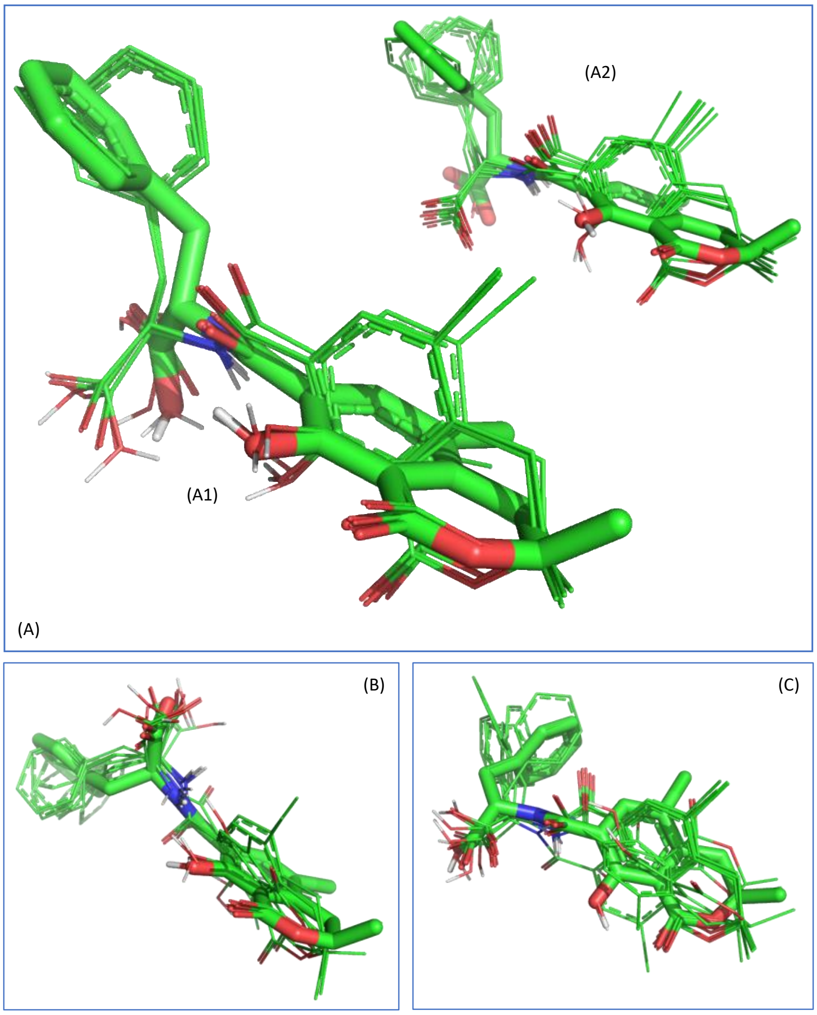

Figure S1. Computer generated views of the aligned energy-minimized 9 states of ochratoxin A (OTA) docking into β-(1,3)-D-glucans chain alone (**A**) with A1 corresponding to OTA with no charge (corresponding to charge state at pH 3.0) and A2 with a partial charge equal to −1 (corresponding to charge state at pH 6.5); into β-(1,3)-D-glucans branched with β-(1,6)-D-glucans conformation 1 (**B**); and conformation 2 (**C**). In these views, only the OTA is represented to account for conformational changes of the ligand molecule.

Table S1. Energy values describing the affinity of interaction between ochratoxin A (OTA) and 3 different β-D-glucans conformations found in yeast cell wall extract.

Receptor Conformation	Mode	Binding Affinity of OTA (OTA-)	Distance from Best Mode for OTA (OTA-)	
		(kcal/mol)	RMSD Lower Bound	RMSD Upper Bound
β-(1,3)-D-glucans	1	−7.8 (−7.8)	0.000	0.000
	2	−7.8 (−7.6)	16.518 (6.029)	17.354 (8.254)
	3	−7.5 (−7.6)	8.058 (15.256)	8.455 (16.367)
	4	−7.5 (−7.6)	8.370 (8.467)	12.131 (12.118)
	5	−7.5 (−7.4)	4.243 (17.300)	6.818 (19.875)
	6	−7.5 (−7.3)	8.772 (2.910)	10.082 (8.297)
	7	−7.4 (−7.1)	2.709 (8.144)	8.284 (8.559)
	8	−7.4 (−7.0)	3.053 (9.230)	8.398 (10.822)
	9	−7.3 (−7.0)	16.183 (16.779)	18.375 (18.898)
β-(1,3)-D-glucans branched with 3× β-(1,6)-D-glucans conformation 1	1	−7.6	0.000	0.000
	2	−7.6	4.809	9.094
	3	−7.6	4.352	6.870
	4	−7.4	3.307	6.662
	5	−7.3	5.152	6.959
	6	−7.0	4.429	6.875
	7	−7.0	7.265	9.729
	8	−7.0	4.595	10.080
	9	−6.9	7.126	9.398
β-(1,3)-D-glucans branched with 3× β-(1,6)-D-glucans conformation 2	1	−7.4	0.000	0.000
	2	−7.3	6.473	10.175
	3	−7.1	4.922	8.050
	4	−7.1	7.098	10.478
	5	−7.1	4.183	7.712
	6	−7.1	5.568	9.246
	7	−7.1	4.093	7.994
	8	−7.0	11.527	14.842
	9	−7.0	3.685	6.188

Root mean square deviation (RMSD) values measuring the average distance between atoms of a position relative to the best fitting position, are calculated using only movable heavy atoms. Two variants of RMSD metrics are provided, rmsd/lb (RMSD lower bound) and rmsd/ub (RMSD upper bound), differing in how the atoms are matched in the distance calculation: rmsd/ub matches each atom in one conformation with itself in the other conformation, ignoring any symmetry. rmsd′ matches each atom in one conformation with the closest atom of the same element type in the other conformation rmsd/lb is defined as follows: rmsd/lb (c1, c2) = max (rmsd′ (c1, c2), rmsd′ (c2, c1)), with cn conformation n.
